# Machine Learning Methods Applied for Modeling the Process of Obtaining Bricks Using Silicon-Based Materials

**DOI:** 10.3390/ma14237232

**Published:** 2021-11-26

**Authors:** Costel Anton, Silvia Curteanu, Cătălin Lisa, Florin Leon

**Affiliations:** 1 Faculty of Chemical Engineering and Environmental Protection, “Gheorghe Asachi” Technical University of Iasi, Str. Prof. Dr. Doc. Dimitrie Mangeron, No. 73, 700050 Iasi, Romania; costel.anton@gmail.com; 2 Faculty of Automatic Control and Computer Engineering, “Gheorghe Asachi” Technical University of Iasi, Str. Prof. Dr. Doc. Dimitrie Mangeron, No. 27A, 700050 Iasi, Romania; florin.leon@academic.tuiasi.ro

**Keywords:** machine learning, neural networks, random forest, bricks, influence of additives

## Abstract

Most of the time, industrial brick manufacture facilities are designed and commissioned for a particular type of manufacture mix and a particular type of burning process. Productivity and product quality maintenance and improvement is a challenge for process engineers. Our paper aims at using machine learning methods to evaluate the impact of adding new auxiliary materials on the amount of exhaust emissions. Experimental determinations made in similar conditions enabled us to build a database containing information about 121 brick batches. Various models (artificial neural networks and regression algorithms) were designed to make predictions about exhaust emission changes when auxiliary materials are introduced into the manufacture mix. The best models were feed-forward neural networks with two hidden layers, having MSE < 0.01 and r^2^ > 0.82 and, as regression model, kNN with error < 0.6. Also, an optimization procedure, including the best models, was developed in order to determine the optimal values for the parameters that assure the minimum quantities for the gas emission. The Pareto front obtained in the multi-objective optimization conducted with grid search method allows the user the chose the most convenient values for the dry product mass, clay, ash and organic raw materials which minimize gas emissions with energy potential.

## 1. Introduction

Due to the numerous external factors, such as the quality of raw materials, the environmental protection requirements that need to be satisfied, and the variations of process parameters, many production processes are complex and extremely energy-and time-consuming. For these reasons, it is not always possible to identify the relation between the product quality and process input variables, hence, the researchers’ effort to integrate artificial intelligence into production processes for data storage, learning, reasoning and decision making.

Neural networks may be used for monitoring, but also for predicting various parameters [1,2]. The existing literature research on brick manufacture includes, for instance, artificial intelligence tools for automating the autoclaved aerated concrete (AAC) brick manufacturing process by creating prototype equipment. The raw material mixing process has been optimized so as to obtain bricks of appropriate hardness, more homogeneous than in the case of their manual manufacture [3]. The relation between the composition of materials which AAC bricks are made from and their mechanical properties was modeled by Zulkifli et al. using neural networks [4]. Other studies use various artificial intelligence tools to develop optimal models for brick-mortar structural composites with improved resistance to elastic deformation [5] or to predict the shear strength of brick masonry walls [6].

In a recent study, Goel and collaborators used regression algorithms, namely random forest to assess the impact of adding degraded municipal solid waste (paper mill sludge, water hyacinth, paper mill sludge compost and water hyacinth compost) in the raw material, in percentages between 0 and 20%, on the functional properties (compressive strength, water absorption and linear shrinkage) of burnt bricks obtained on a laboratory scale. The authors established that up to 15% of organic solid waste leads to a compressive strength comparable to normal clay brick. They also found that degraded municipal solid waste is the optimal additive to be used to achieve maximum compressive strength and minimum water absorption [7].

The use of different types of waste in the industry for obtaining burnt bricks has important benefits related to both significant savings in raw materials [8] and energy [9] in the combustion process. The use of solid waste such as sawdust [10], ash [11,12,13], agricultural waste [14,15], paper waste [16], textiles [17], marble stone powder [8] and others [18,19,20,21] in the manufacture of burnt bricks has been tested on a laboratory scale. Cultrone and others [10] have shown in their study that adding sawdust to the brick manufacturing mix does not change their mineralogy, making them lighter and better thermal insulators. They also established that they can be burned at lower temperatures, significantly reducing energy costs. High burning temperatures did not improve the insulating properties of sawdust-containing bricks in the manufacturing mix.

The documentation of the authors of this paper has not identified applications of artificial intelligence tools in assessing the impact of changing the composition of raw material used for brick manufacture on the content and amount of pollutants imposed by legal environmental protection regulations.

Burnt clay products have been used as building materials since ancient times. The process of obtaining them includes several stages that contribute to obtaining the finished product: (1) obtaining an optimal manufacturing mix characterized by stability and repeatability over time, whose properties influence the design of the facilities in the following stages; (2) modeling products by pressing or shaping; (3) drying them in room-like or tunnel-like dryers; (4) burning of products by heating them to high temperatures in order to provide them with mechanical strength, resistance to freeze/thaw cycles (durability) and resistance to softening under the action of water. In recent years, brick burning has taken place in tunnel kilns which allow large-scale daily output, but which require the existence of stable processes that, once determined, do not require any changes. Each burning regime change of tunnel kilns causes significant disruptions to the technological process and slowing down production. Most of the times, facilities are designed for a particular type of manufacturing mix and a particular type of burning process, then, for different reasons, the initial conditions, designed and implemented, later change radically: natural gas costs, raw material costs, the need to increase productivity, changing the accepted limits in the area of flue gases. There is a need to maintain and improve productivity, performance and manufacturing costs of products. Thus, new mixtures are fed into the initial equipment and virtually the same characteristics of the mix (drying/burning shrinkage), of the drying curve (ability to lose physically bound water in the unit of time) and burning curves must be observed due to the constructive characteristics of the facility. This may cause costs increase, waste, pollution etc. The avoid such problems many hours of test-mode production with high costs and uncertain results are necessary. Therefore, in this paper we suggest a new approach to the impact of changes in the burning process while keeping the original facility and altering the manufacturing mix. The possibility of studying the process by simulation is extremely advantageous due to the information that can be collected without conducting costly experiments. In previous studies in which the industrial process of wastewater purification in aerated lagoons was analyzed [22,23], models that can replace experiments, saving time, material and energy, were proposed.

This experimental study consists in several stages. First, a statistical analysis is applied for evaluating the distribution of experimental data and selecting the input parameters that significantly influence the considered output parameters. Then, neural network models and other regression methods [24] are used in order to find the most appropriate one that can provide a good approximation for the data. Secondly, after the modeling step, a multi-objective optimization is performed to find the most appropriate values for the inputs (i.e., dry product mass, clay, ash and organic raw materials) that lead to the minimum values of the outputs (i.e., CO, NO and CH_4_). Because the dimensionality of the problem is not too big, for this multi-objective optimization, a grid search approach is applied. The obtained results are satisfactory and in good agreement with the experimental practice.

## 2. Materials and Methods

### 2.1. Experimental Setting

The impact of adding auxiliary materials on the burning performance in an industrial brick burning facility was assessed by analyzing the exhaust gases in the furnace chimney. Burning performance is defined as the sum of the characteristics that have in view the preservation of the physical-mechanical properties of the bricks, the reduction of costs and keeping the previous technological flow unchanged, from the formation-extrusion area to the finished/burned product area. The combustion process took place in a tunnel oven by heating the dry products to high temperatures. All the technological features were kept constant: the same amount of product, the same combustion rate, the same combustion curve settings and the same exhaust gas sampling location. Only the type of the used auxiliary raw material of an organic nature was changed: sawdust and sunflower seed husks. In the manufacturing mix, there were used 15% ash and various percentages of sunflower seed husks or sawdust (0 and 3.5%) for 121 brick production batches and the impact on the noxious substances emitted from the furnace chimney was evaluated. The gases exhausted by the furnace chimney were analyzed using a Testo 350 flue gas analyzer (Testo, Titisee-Neustadt, Germany) equipped with detection and measurement cells specific to those gases (CO, NO_x_, C_x_H_y_), metrologically calibrated. The analyzer’s CO measuring resolution is 0.1 ppm, that of NO is 1 ppm, for NO_2_ is 0.1 ppm and for C_x_H_y_ is 1 ppm. For these parameters and for this type of equipment, other researchers reported a measurement uncertainty of ±2.51% [25] and ±5% [26]. For the accuracy of the results, a series of determinations were performed on different days of operation, by keeping constant the other parameters, for 15 min, with readings from minute to minute. The results taken into account were the arithmetic mean of the 15 readings. The same sampling location and length of the sampling probe were also maintained so that we can consider that the same measurement conditions for the exhaust gases at the furnace chimney were present. The uncertainties calculated for CO were ±7.7%, for NO 9.5% and for CH_4_ 8.4%. Acceptable emission limits imposed by the local environmental agency are: NO_x_ < 250 mg/m^3^; CO < 1500 mg/m^3^ and volatile organic compounds <20 mg/m^3^.

Based on the experimental measurements performed on dry product mass, number of pieces/kiln car, total tons/day, amount of clay, amount of ash, amount of organic raw materials and values of pollutants measured in the chimney (CO, NO and CH_4_), a database containing information on 121 batches of bricks was developed.

### 2.2. Statistical, Modeling and Optimization

The statistical processing of the available experimental data was performed with the specialized SigmaPlot 11.00 software (Systat Software Inc., San Jose, CA, USA). The information related to the average values, standard deviation, standard mean error, confidence interval of the average, amplitude, maximum value, minimum value, median, distribution ranges of 25% and 75% of data and evaluation of normal data distribution was provided: skewness and kurtosis tests, Kolmogorov-Smirnov test, Shapiro-Wilk test, the sum of the data and the sum of the squares which is a measure of the deviation from the average value.

**Figure 1:**
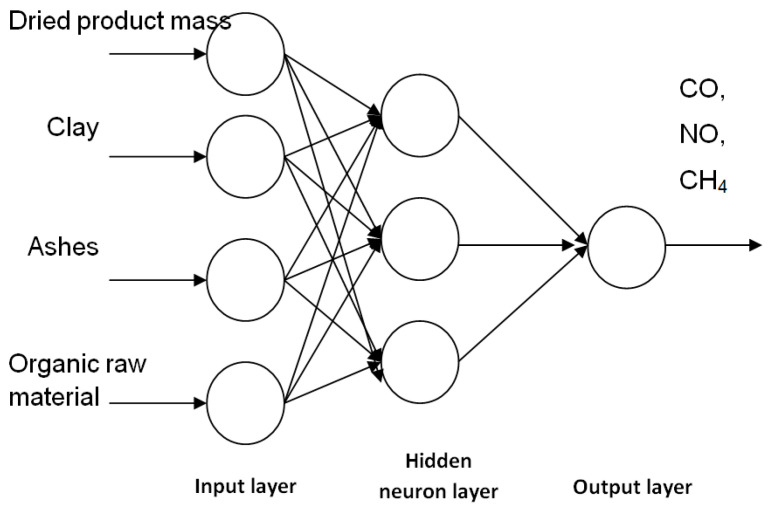
Neural network structure.

The NeuroSolutions specialized software (NeuroDimension Inc., Boston, MA, USA) was used to build neural models as forward propagation networks. The experimental data provide information about 121 batches of bricks. 100 of them were used in the neural network training stage and 21 were kept for the testing stage. In order to establish the topology of the artificial neural networks (ANNs) with the best possible results, several networks of the type shown in Figure 1 were tested, with 4 inputs, one or two hidden layers with 4 to 80 hidden neurons and an output for predicting the amount of CO, NO and CH_4_ exhausted in the flue gas chimney.

Other regression algorithms were also used for modeling. Nearest neighbor (NN) and k-nearest neighbor (kNN) are instance-based algorithms [27], where the predicted value of a query instance is computed either as the value of the closest training point according to the Euclidian distance metric, or as a weighted average taking into account k such closest neighbors, where their weights are set as a function of the distance between the query instance and the corresponding training instance. For example, many times the inverse distance function is used: w_i_ = 1/d_i_.

The K* algorithm [28] has a similar philosophy, but it uses entropy as a distance metric, justified by the idea that the distance between two instances can be defined as the complexity of transforming one instance into the other. It uses a global blend (gb) parameter, which can be considered as a sphere of influence that implicitly specifies how many neighbors are significant.

Support vector regression (SVR) is a method inspired from support vector machines classification [29], whose idea is the minimize the error by finding output values that lie within a given margin named an ε-tube. The objective is to maximize the number of points that can be placed inside this tube, i.e., within the margin. Different kernels can be applied to the data to transform them into a configuration easier to learn, e.g., polynomial of different degrees, radial basis function (RBF) or Pearson universal kernel (PUK). When after such transformation the data still cannot be fit completely into the ε-tube, some errors are allowed and the cost parameter C controls the strictness of the objective function that is optimized by the algorithm: a higher value for C will lead to a smaller margin with a lower error, while a lower value of C will allow a greater error on the training set but with a wider margin, which in turn may lead to better generalization capabilities. Random Forest [30] is an ensemble method where a forest is composed of a set of trees. The trees are constructed by recursively choosing a partition after an attribute from a random subset of attributes. Also, each tree is built on a slightly different dataset using bagging, i.e., sampling from the initial dataset uniformly with replacement. In this way, the trees are sufficiently diverse to capture different perspectives of the training set. For a query instance, each tree computes an output value and the random forest ensemble calculates the average of these individual values.

## 3. Results

### 3.1. Statistical Processing of Experimental Data

Table 1 presents statistical description of the data. We find lower values of the standard deviation for the dry product mass (Col 1) and the quantity of organic raw materials (Col 6), which indicates a lower spread of these experimental data. The amplitude, respectively the difference between the maximum and the minimum value, which indicates the range of values in which the distribution of experimental data extends, has values between 8 and 963. The median, the statistical parameter that indicates the middle of the data series, as long as it is organized in ascending or descending direction, has values close to the average value which shows a uniform distribution of experimental data. The analysis of the experimental data distribution reveals a negative asymmetry for Col 1–6 and a positive one for Col 7–9.

The flattening indices of the variation curve of the analyzed data (kurtosis) have small values, which indicates a good distribution of the data, i.e., the fact that there are few data that have values very different from the average. The Kolmogorov-Smirnov normality test, which quantifies the degree of overlap between the cumulative distribution of the analyzed variables and the cumulative distribution of the variable following the shape of the Gaussian curve, indicates a normal distribution of data in the case of noxious substances measured in the chimney: Col 7 (CO mg/m^3^) and Col 9 (CH_4_ mg/Nm^3^). This is also confirmed by the results obtained with the Shapiro-Wilk normality test.

In order to design neural models that link a series of selected parameters regarding the pollutants released in a brick factory to the raw materials used, the statistical processing of the available experimental data was performed first.

To see if there are statistically significant differences between the 9 pairs of data series, the Kruskal-Wallis test was applied, namely the multiple pair comparison procedures (Dunn’s Method). According to the Q values from the Dunn’s test, presented in Table 2, it was established that there are no statistically significant differences (*p* > 0.05) between the total tons (Col 3) and the amount of CO (Col 7); between the total tons (Col 3) and the amount of clay (Col 4); between the quantity of organic raw materials (Col 6) and the dry product mass (Col 1); between the amount of clay (Col 4) and the amount of CH4 (Col 9), and between the amount of ashes (Col 5) and the amount of NO (Col 8).

Following the statistical processing of the experimental data and for practical reasons, the following data series were used for modeling with neural networks: dry product mass (Col 1), quantity of clay (Col 4), quantity of ashes (Col 4), organic raw materials (Col 4), and the values of noxious substances measured in the chimney: CO, NO, and CH_4_ (Col 7, Col 8 and Col 9).

### 3.2. Neural Network Modeling

The mean square error (MSE), correlation coefficient (r^2^) and percentage error Ep (%) were used as criteria for choosing the best topology. Network topology was encoded by (m:n:p), where m is the number of neurons in the input layer, n—the number of neurons in the hidden neuron layer, and p—the number of neurons in the output layer.

In order to avoid the overtraining of the chosen neural networks, the variation of the MSE error with the number of training epochs was analyzed and it was concluded that when the number of epochs exceeds 70,000, the performance stops improving. Therefore, the number of training epochs used was 70,000 for all neural models developed.

**Figure 2:**
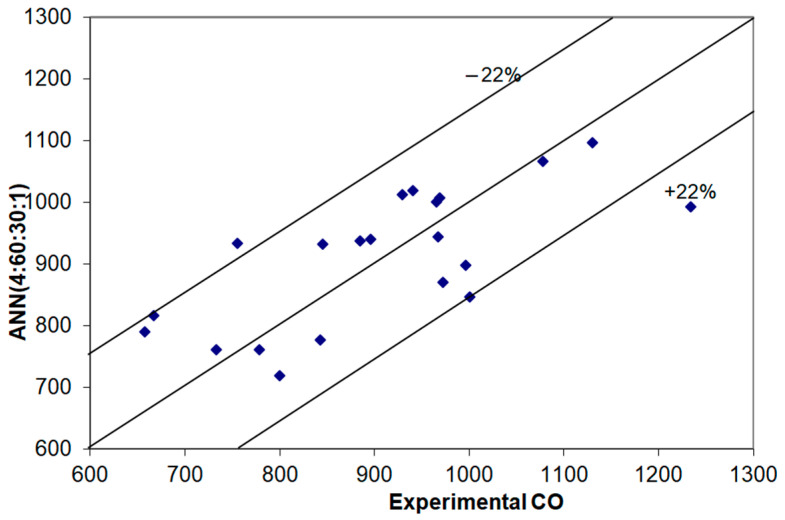
Comparing experimental and simulation results obtained in the validation stage to predict CO.

In order to predict the amount of CO exhausted in the flue gas chimney, neural models with the MSE, r^2^ and Ep topologies and errors shown in Table 3 were used. According to Figure 2, almost all results are within a ±22% confidence interval.

**Figure 3:**
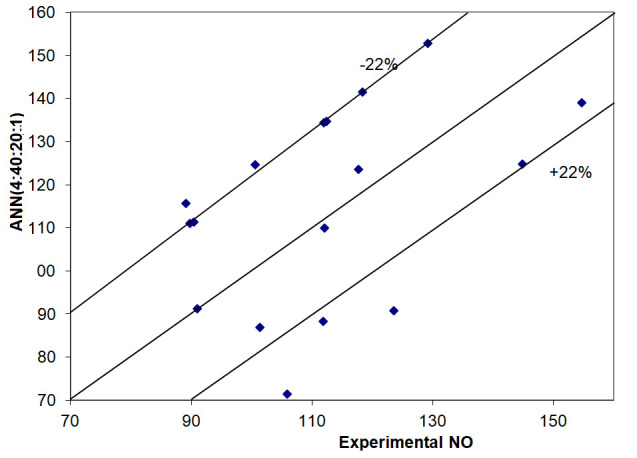
Comparing experimental and simulation results obtained in the validation stage to predict NO.

**Figure 4:**
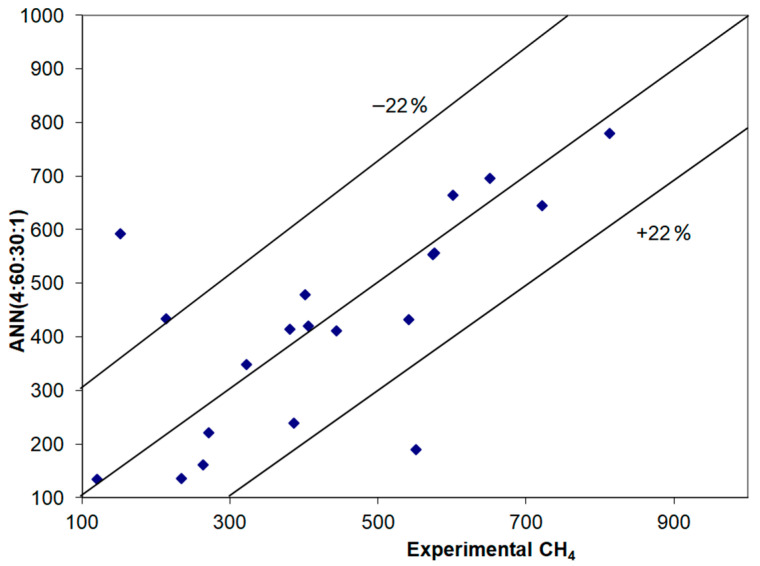
Comparing experimental and simulation results obtained in the validation stage to predict CH_4_.

Table 4 and Table 5 show the neural models developed to predict the amount of NO and CH_4_, respectively, exhausted in the flue gas chimney. The best performance was achieved in the training stage using the ANN (4:40:20:1) model for NO and ANN (4:60:30:1) model for CH_4_. Figure 3 and Figure 4 compare the results achieved using these models with the experimental ones and the conclusion is that for the most cases, the confidence interval was of these figures are also within the ±22% confidence interval, with the exception of three values for NO and two values for CH_4_.

The possibility to make predictions regarding the presence in the flue gases of the quantity of gases with energy potential (CH_4_), and also of the quantity of polluting gases (NO_x_ and CO) allow decision makers to decide whether to invest in facility retrofitting or to change certain operating limits so as to keep process and product performance in the comfort zone, while improving costs.

### 3.3. Modeling with Regression Methods

Table 6 shows the regression results for each of the three outputs of the problem (CO, NO and CH_4_) applying different algorithms and combinations of parameter values. The results are presented in terms of the correlation coefficient (r), where a value closer to 1 designates a better data fit. For each variant of the algorithm, the results for the training set and for 10-fold cross-validation (CV) are displayed. However, we are interested in good generalization, therefore the CV results are used to select the best models. The best values obtained for each of the three outputs are marked in bold.

As one can see, there is no single best model for all three outputs. Still, k-nearest neighbor and random forest are the algorithms that stand out at the most promising for this problem. The second variant of kNN (k = 10) is better for CO, but much worse for NO than kNN (k = 6). Thus, kNN (k = 6, w = 1/d) and random forest (1000 trees) were selected for the second step of the optimization, together with a combination of models, a separate one for each output.

Indeed, the results of cross-validation are not very good, but can be considered satisfactory and useful. What is very important to emphasize in this approach is that the experiments were performed in industrial conditions, which means very large amounts of time, materials and energy per batch. Hence, the limited number of experiments which, together with the accuracy of the experimental determinations, influence the modeling results. Even in these conditions, the results obtained are of real use to industrial practice through the indications provided regarding the composition of the materials used.

### 3.4. Process Optimization

In the second stage, the model found previously is used for optimization, i.e., finding the most appropriate values for the inputs (dry product mass, clay, ash and organic raw materials) that lead to the minimum values of the outputs (CO, NO and CH_4_). Since the outputs are more than one, it leads to a multi-objective optimization problem. Specialized algorithms exist for this type of problems, e.g., a widely used evolutionary algorithm is NSGA-II [31]. An evolutionary algorithm is very useful to explore the problem space, especially when the dimensionality of the problem is big. However, in our case, the problem has only four attributes. Since a short execution time is not a requirement here, and we are more interested in the quality of the solution, we chose a grid search approach instead. 

In this case, the space problem is explored on each dimension with a proportional step. Let *x_min_* and *x_max_* be the minimum and maximum values of input *x*. The number of steps *s*, which determines the resolution on the axis, is defined by the user. In our case, *s* = 50 for all inputs. The step size is therefore *s_size_* = (*x_max_* − *x_min_*)/*s*. This leads to a number of 50^4^ = 6,250,000 points (a large, but tractable amount) for which the outputs are computed.

From these triplets (CO_i_, NO_i_, CH_4i_), the non-dominated solutions are identified. A potential solution *i* dominates another potential solution *j* if *i* is better or equal to *j* for all objectives, and strictly better than *j* for at least one objective. Because the Pareto front is a 3D surface, a 2D (*x*, *y*) plot was used with the *z* axis represented by color.

**Figure 5:**
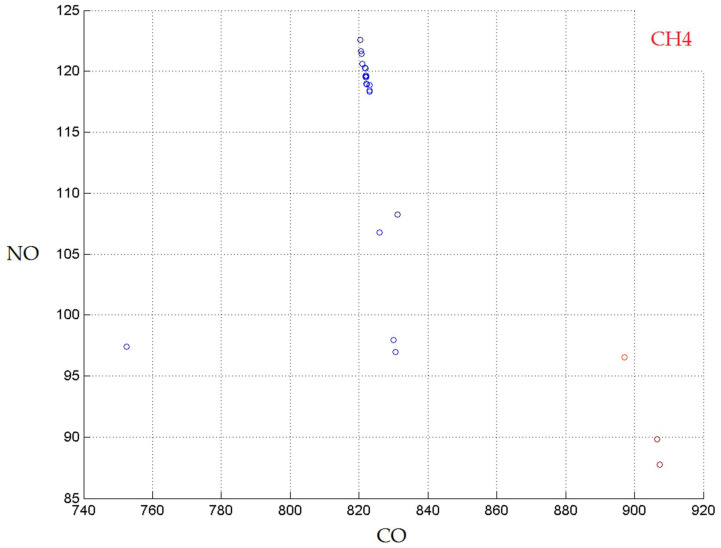
The Pareto front obtained with the kNN models.

The results of the optimization, i.e., the Pareto front, using the kNN (k = 6, w = 1/d) models are presented in Figure 5.

**Figure 6:**
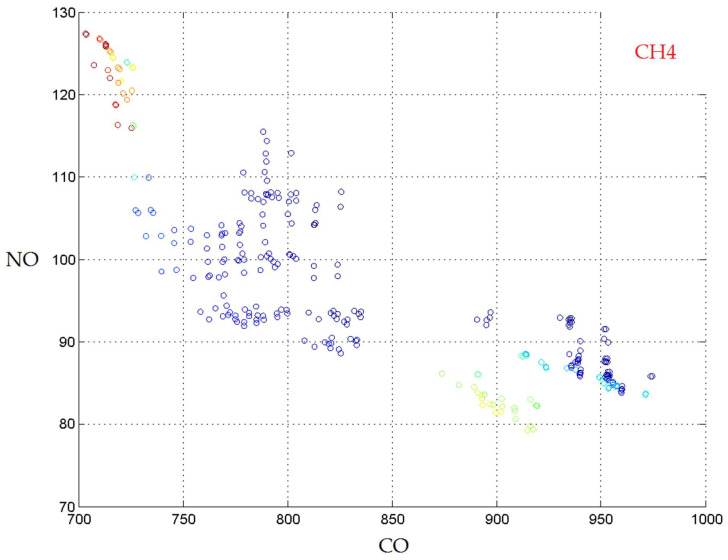
The Pareto front obtained with the Random Forest models.

The Pareto front using the Random Forest (1000 trees) models is presented in Figure 6.

**Figure 7:**
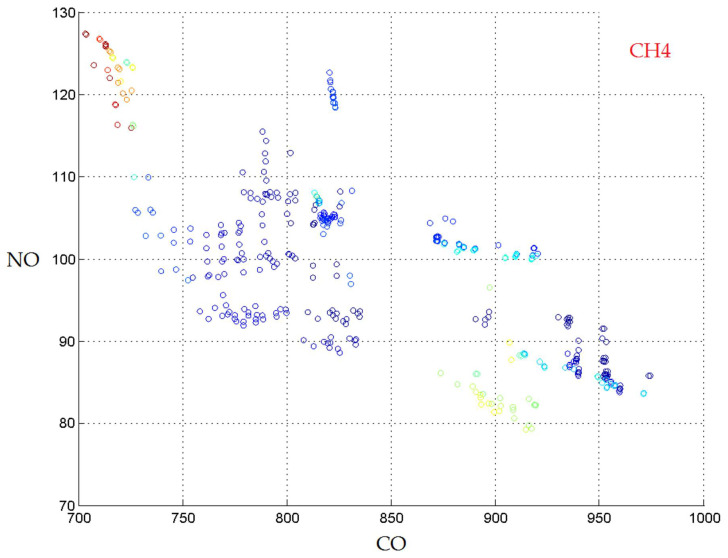
The Pareto front obtained with three separate models.

Figure 7 displays the Pareto front obtained for three separate models with kNN(k = 10, w = 1/d), kNN (k = 6, w = 1/d) and random forest (1000 trees) for the outputs CO, NO, and CH_4_, respectively. They are the models which yielded the best individual results, as shown in Table 6.

The last two fronts are much more diverse than the first. Still, all three fronts contain non-dominated solutions and the actual combination of reaction conditions should be chosen by the practitioner according to the estimated relative importance of the outputs. The obtained results reveal that no dominant solutions were obtained so that we can say that all the considered input parameters (dry product mass, clay, ash and organic raw materials) significantly influence the values of the output parameters (CO, NO and CH_4_). For the prediction of CO and CH_4_, which are gas emissions with energy potential, discharged to the furnace, the best correlation coefficients with kNN (k = 10) and Random Forest (1000 trees) were obtained, respectively: 0.6355 and 0.7384. These values are slightly lower than those reported by other authors in the literature, but which used regression algorithms, namely Random Forest regression to assess the impact of adding solid organic waste in the raw material used to obtain burnt bricks (on a laboratory scale) on their functional properties [7]. We consider that the use of the models developed in our study to predict the amount of CO and CH_4_ can provide very useful information for process engineers, because they use experimental data obtained in an industrial plant in real working conditions in which up to 90,000 of pieces of bricks a day are obtained. It is possible to evaluate, with the help of the elaborated models, the impact of the modification of the composition of the raw material used for the manufacture of the bricks on the quantity of noxious substances that are evacuated to the furnace basket. In order to comply with the limits imposed by legal regulations for environmental protection, it is possible to reduce the number of test batches with different mixtures of raw materials which shall lead to maintaining and improving product performance on the same plant, respecting the same combustion curves in advantageous economic conditions.

## 4. Conclusions

This study approaches a real problem of industrial practice, i.e., trying to evaluate the impact of adding auxiliary materials (sawdust and sunflower seed husks) on the burning performance in an industrial brick burning facility. The research was conducted experimentally and by simulation.

Based on the experimental measurements performed on dry product mass, number of pieces/kiln car, total tons/day, amount of clay, amount of ash, amount of organic raw materials and values of pollutants measured in the chimney (CO, NO and CH_4_), a database containing information on 121 batches of bricks was developed.

The simulation methods applied here include artificial neural networks and regression algorithms (nearest neighbor, k-nearest neighbor, support vector regression, random forest) for modeling and a grid search for multi-objective optimization.

The best models were feed-forward neural networks with two hidden layers, having MSE < 0.01 and r^2^ > 0.82 and, as regression models, kNN with error < 0.6. 

These models were included into the optimization procedure to determine the working conditions (i.e., dry product mass, clay, ash and organic raw materials) that lead to the minimum quantities of gas emission (i.e., CO, NO and CH_4_). For the prediction of CO and CH_4_, which are gas emissions with energy potential, discharged to the furnace, the best correlation coefficients with kNN (k = 10) and random forest (1000 trees) were obtained, i.e., 0.6355 and 0.7384.

The results obtained by simulation are useful for industrial practice replacing expensive experiments that consume significant resources of time, energy and materials.

In addition, the paper intends to present a new working methodology, a study by simulation based on tools provided by artificial intelligence, a method that can be applied under different conditions and on different data sets.

## Figures and Tables

**Table 1:** Statistical description of experimental data.

**Column**	**Size**	**Missing**	**Mean**	**Std. Dev.**	**Std. Error**	**C.I. of Mean**	**Range**	**Max**	**Min**	**Median**
Col 1	121	0	14.885	3.219	0.293	0.579	8.49	18.7	10.21	14.11
Col 2	121	0	1538.471	303.036	27.549	54.545	2071	2205	134	1344
Col 3	121	0	739.899	131.261	11.933	23.626	813.137	889.85	76.712	785.862
Col 4	121	0	606.701	107.98	9.816	19.436	667.156	729.677	62.521	644.097
Col 5	121	0	110.985	19.689	1.79	3.544	121.971	133.477	11.507	117.879
Col 6	121	0	22.213	4.502	0.409	0.81	26.247	28.932	2.685	23.406
Col 7	121	0	914.885	123.356	11.214	22.203	576.052	1233.708	657.656	913.87
Col 8	121	0	112.32	22.238	2.022	4.003	99.137	166.258	67.121	109.788
Col 9	121	0	395.969	205.602	18.691	37.007	962.734	979.563	16.828	384.978
**Column**	**25%**	**75%**	**Skewness**	**Kurtosis**	**K-S Dist.**	**K-S Prob.**	**SWilk W**	**SWilk Prob.**	**Sum**	**Sum of Squares**
Col 1	11.483	17.982	−0.178	−1.705	0.273	<0.001	0.818	<0.001	1801.1	28,053.031
Col 2	1344	1890	−0.0657	2.74	0.318	<0.001	0.745	<0.001	186155	297,413,803
Col 3	685.73	822.174	−2.108	5.76	0.213	<0.001	0.779	<0.001	89,527.831	68,309,121
Col 4	563.507	677.172	−2.085	5.668	0.199	<0.001	0.783	<0.001	73,410.829	45,937,579
Col 5	102.859	123.326	−2.108	5.76	0.213	<0.001	0.779	<0.001	13,429.175	1,536,955.2
Col 6	20.334	25.153	−1.179	2.404	0.152	<0.001	0.916	<0.001	2687.827	62,137.641
Col 7	823.385	992.977	0.303	−0.298	0.0514	0.564	0.987	0.281	110,701.05	103,104,680
Col 8	94.526	129.049	0.389	−0.59	0.0948	0.01	0.97	0.009	13,590.734	1,585,858.2
Col 9	237.99	540.703	0.362	−0.396	0.0515	0.56	0.982	0.097	47,912.303	24,044,489

Col 1(Dry product mass, kg); Col 2 (No. piece/kiln car); Col 3 (Total tons/day); Col 4 (clay, tons); Col 5 (Ash tons); Col 6 (Organic raw materials, tons); Values of the noxious substances measured in the chimney: Col 7 (CO mg/m^3^); Col 8 (NO mg/m^3^); Col 9 (CH_4_ mg/Nm^3^).

**Table 2:** Results of the Kruskal-Wallis test.

Comparison	Diff. of Ranks	Q	*p* < 0.05
Col 2 vs. Col 1	952	23.544	Yes
Col 2 vs. Col 6	851.777	21.065	Yes
Col 2 vs. Col 8	653.686	16.166	Yes
Col 2 vs. Col 5	648.62	16.041	Yes
Col 2 vs. Col 9	463.57	11.465	Yes
Col 2 vs. Col 4	362.86	8.974	Yes
Col 2 vs. Col 3	248.603	6.148	Yes
Col 2 vs. Col 7	138.215	3.418	Yes
Col 7 vs. Col 1	813.785	20.126	Yes
Col 7 vs. Col 6	713.562	17.647	Yes
Col 7 vs. Col 8	515.471	12.748	Yes
Col 7 vs. Col 5	510.405	12.623	Yes
Col 7 vs. Col 9	325.355	8.046	Yes
Col 7 vs. Col 4	224.645	5.556	Yes
Col 7 vs. Col 3	110.388	2.73	No
Col 3 vs. Col 1	703.397	17.396	Yes
Col 3 vs. Col 6	603.174	14.917	Yes
Col 3 vs. Col 8	405.083	10.018	Yes
Col 3 vs. Col 5	400.017	9.893	Yes
Col 3 vs. Col 9	214.967	5.316	Yes
Col 3 vs. Col 4	114.256	2.826	No
Col 4 vs. Col 1	589.14	14.57	Yes
Col 4 vs. Col 6	488.917	12.091	Yes
Col 4 vs. Col 8	290.826	7.192	Yes
Col 4 vs. Col 5	285.76	7.067	Yes
Col 4 vs. Col 9	100.711	2.491	No
Col 9 vs. Col 1	488.43	12.079	Yes
Col 9 vs. Col 6	388.207	9.601	Yes
Col 9 vs. Col 8	190.116	4.702	Yes
Col 9 vs. Col 5	185.05	4.576	Yes
Col 5 vs. Col 1	303.38	7.503	Yes
Col 5 vs. Col 6	203.157	5.024	Yes
Col 5 vs. Col 8	5.066	0.125	No
Col 8 vs. Col 1	298.314	7.378	Yes
Col 8 vs. Col 6	198.091	4.899	Yes
Col 6 vs. Col 1	100.223	2.479	No

Col 1(Dry product mass, kg); Col 2 (No. piece/kiln car); Col 3 (Total tons/day); Col 4 (clay, tons); Col 5 (Ash tons); Col 6 (Organic raw materials, tons); Values of the noxious substances measured in the chimney: Col 7 (CO mg/m^3^); Col 8 (NO mg/m^3^); Col 9 (CH_4_ mg/Nm^3^).

**Table 3:** Topology of various ANN forward propagation networks developed to predict CO.

No.	Topology	MSE	r^2^	E_p_ (%)
1.	ANN (4:4:1)	0.0199	0.696	7.18
2.	ANN (4:8:1)	0.0181	0.728	6.58
3.	ANN (4:12:1)	0.0177	0.735	6.56
4.	ANN (4:16:1)	0.0175	0.739	6.50
5.	ANN (4:20:1)	0.0177	0.735	6.50
6.	ANN (4:40:1)	0.0165	0.757	6.11
7.	ANN (4:40:20:1)	0.0138	0.801	4.92
8.	ANN (4:60:30:1)	0.0123	0.825	4.76
9.	ANN (4:80:40:1)	0.0126	0.821	4.77

**Table 4:** Topology of various ANN forward propagation networks developed to predict NO.

No.	Topology	MSE	r^2^	E_p_ (%)
1.	ANN (4:4:1)	0.0241	0.547	14.08
2.	ANN (4:8:1)	0.0230	0.665	11.91
3.	ANN (4:12:1)	0.0260	0.610	13.21
4.	ANN (4:16:1)	0.0290	0.529	14.42
5.	ANN (4:20:1)	0.0229	0.609	11.69
6.	ANN (4:40:1)	0.0232	0.664	12.03
7.	ANN (4:40:20:1)	0.0082	0.895	5.88
8.	ANN (4:60:30:1)	0.0138	0.816	7.84
9.	ANN (4:80:40:1)	0.0117	0.847	6.68

**Table 5:** Topology of various ANN forward propagation networks developed to predict CH_4_.

No.	Topology	MSE	r^2^	E_p_ (%)
1.	ANN (4:4:1)	0.0114	0.835	51.07
2.	ANN (4:8:1)	0.0093	0.867	41.25
3.	ANN (4:12:1)	0.0090	0.872	40.67
4.	ANN (4:16:1)	0.0092	0.869	40.91
5.	ANN (4:20:1)	0.0090	0.872	39.43
6.	ANN (4:40:1)	0.0094	0.865	43.36
7.	ANN (4:40:20:1)	0.0074	0.895	34.90
8.	ANN (4:60:30:1)	0.0070	0.902	33.63
9.	ANN (4:80:40:1)	0.0073	0.897	34.47

**Table 6:** The results obtained with several regression algorithms.

Algorithm	Dataset	CO	NO	CH_4_
kNN (k = 6, w = 1/d)	Training	0.9261	0.9251	0.9525
	Cross-validation	0.6315	0.5184	0.7203
kNN (k = 10, w = 1/d)	Training	0.8127	0.7336	0.7790
	Cross-validation	0.6355	0.4666	0.7081
NN (k = 1)	Training	0.9978	0.9893	0.9875
	Cross-validation	0.4927	0.4371	0.6473
K* (gb = 10)	Training	0.9392	0.9456	0.9641
	Cross-validation	0.5954	0.4529	0.6449
K* (gb = 20)	Training	0.8945	0.8927	0.9359
	Cross-validation	0.6295	0.4608	0.6840
K* (gb = 50)	Training	0.7924	0.7540	0.8670
	Cross-validation	0.6061	0.4461	0.7197
SVR (C = 10,000, PUK)	Training	0.7999	0.7812	0.8841
	Cross-validation	0.2349	0.1995	0.1880
SVR (C = 100, poly d = 2)	Training	0.6443	0.3835	0.7736
	Cross-validation	0.5938	0.1619	0.7061
SVR (C = 100, RBF)	Training	0.6277	0.3061	0.6432
	Cross-validation	0.6037	0.2103	0.5999
Random Forest(100 trees)	Training	0.9592	0.9418	0.9621
	Cross-validation	0.5994	0.4799	0.7120
Random Forest(1000 trees)	Training	0.9613	0.9468	0.9612
	Cross-validation	0.6045	0.4878	0.7384

kNN—k-Nearest Neighbors; k = the number of neighbors; wi = the weight of instance i; di = the distance from the query point to instance i; NN = Nearest Neighbor; gb = global blend; SVR = Support Vector Regression; C = the cost parameter; PUK = Pearson Universal Kernel; d = the degree of the polynomial kernel; RBF = Radial Basis Function kernel.
